# Plasma Thermogram Parameters Differentiate Status and Overall Survival of Melanoma Patients

**DOI:** 10.3390/curroncol30070453

**Published:** 2023-06-24

**Authors:** Taylor Q. Nguyen, Gabriela Schneider, Alagammai Kaliappan, Robert Buscaglia, Guy N. Brock, Melissa Barousse Hall, Donald M. Miller, Jason A. Chesney, Nichola C. Garbett

**Affiliations:** 1 UofL Health–Brown Cancer Center and Division of Medical Oncology and Hematology, Department of Medicine, University of Louisville, Louisville, KY 40202, USA; 2 Department of Mathematics and Statistics, Northern Arizona University, Flagstaff, AZ 86011, USA; 3 Department of Biomedical Informatics, College of Medicine, The Ohio State University, Columbus, OH 43210, USA

**Keywords:** differential scanning calorimetry (DSC), thermogram, melanoma, diagnosis, overall survival (OS), no evidence of disease (NED)

## Abstract

Melanoma is the fifth most common cancer in the United States and the deadliest of all skin cancers. Even with recent advancements in treatment, there is still a 13% two-year recurrence rate, with approximately 30% of recurrences being distant metastases. Identifying patients at high risk for recurrence or advanced disease is critical for optimal clinical decision-making. Currently, there is substantial variability in the selection of screening tests and imaging, with most modalities characterized by relatively low accuracy. In the current study, we built upon a preliminary examination of differential scanning calorimetry (DSC) in the melanoma setting to examine its utility for diagnostic and prognostic assessment. Using regression analysis, we found that selected DSC profile (thermogram) parameters were useful for differentiation between melanoma patients and healthy controls, with more complex models distinguishing melanoma patients with no evidence of disease from patients with active disease. Thermogram features contributing to the third principal component (PC3) were useful for differentiation between controls and melanoma patients, and Cox proportional hazards regression analysis indicated that PC3 was useful for predicting the overall survival of active melanoma patients. With the further development and optimization of the classification method, DSC could complement current diagnostic strategies to improve screening, diagnosis, and prognosis of melanoma patients.

## 1. Introduction

Staging and recurrence risk is heavily dependent on the initial presentation of melanoma (i.e., tumor thickness, distant metastasis, lymph node involvement). The American Joint Committee on Cancer (AJCC) and National Comprehensive Cancer Network (NCCN) guidelines provide a framework in which linear clinical decision-making can be performed. Yet, there is discrepancy within each stage, and there is substantial variability in the timing and selection of follow-up laboratory tests and imaging [1,2]. Dinnes et al. conducted a meta-analysis on imaging studies for prognostication and recurrence [3]. They employed stringent exclusion criteria but found that for initial staging, while the specificities of ultrasound, CT, and PET-CT were relatively high (80–90%), the sensitivity of these techniques was low (23–42%). For re-staging, PET-CT had both high sensitivity and specificity (>89% for both) which contributed to higher clinical suspicion for recurrent disease [3].

Several biomarkers or techniques detecting specific chromosomal sequence abnormalities have been proposed for melanoma susceptibility, prognosis, and recurrence. These include SNPs of metabolic pathway genes [4], CD28/CTL4/ICOS genes [5], the Tyrosinase gene [6], VDR genes [7], and PI3K genes [8]. This has allowed the definition of several SNP signatures for melanoma prognosis [9,10]. Another is the preferentially expressed antigen in melanoma (PRAME) that was found to be expressed in 87–92% of metastatic melanomas, but, as a qualitative biomarker, it requires expert interpretation [10].

Some genomic abnormalities detected using fluorescent in situ hybridization (FISH) and comparative genome hybridization (CGH) methods have also been evaluated [10]. Out of two sets of four specific chromosome sequences (6p23, 6p25, 11q13, and centromere 6 or 9p21, 6p25, 11q13, and 8q24), the second set of sequences resulted in sensitivity and specificity of 94% and 98%, respectively, but there is concern that the detection technique (FISH) is expensive and requires specific tools and equipment that not all hospitals can accommodate [10]. DNA copy number changes, evaluated using CGH, seem to be promising in classifying melanocytic tumors, as the technique has shown relative sensitivity and specificity of 96% and 87%, respectively, but the method not only requires large amounts of tissue for analyses but also specific clinical gestalt by dermatopathologists that can lead to significant variability in the interpretation of results [11]. More recently, the Myriad myPath quantitative gene expression panel (GEP; evaluating 23 genes) and scoring algorithm were reported to have favorable test characteristics (sensitivity of 90% and specificity of 91%) [12,13]; however, additional studies with long-term follow-ups of clinical outcomes are needed to determine the utility of this test in clinical practice.

Several biomarkers have also been investigated for the diagnosis of pigmented lesions, mostly assessing the levels of specific proteins, such as Ki-67, p16, and HMB45 [14], or specifically modified DNA, such as 5-hydroxymethylcytosine (5-hmC) [15]. While high sensitivity and specificity (>95%) have been reported, the results require verification on larger cohorts, as well as the standardization of the results of stain scoring used in the test procedure, which is notoriously subjective. Ultimately, further studies need to be conducted to validate the current analytical techniques [14,15].

BRAF mutations, MEK mutations, and programmed cell death protein-1 (PD-1) are specific gene or protein receptor targets that have significantly changed the overall survival (OS) of patients with advanced-stage melanoma. Approximately 50% of melanoma patients have an activating mutation in the BRAF gene [16]. Although these mutations have been associated with poorer prognosis and OS in the past [17], the advent of effective BRAF and MEK inhibitors has resulted in marked improvement in OS [17]. Also, PD-1 inhibitors have demonstrated a robust response in patients that have PD-L1 expression [18]. Yet, these targeted therapies have faced some challenges and difficulties. For example, 15% of patients undergoing BRAF therapy do not respond to treatment and about 50% develop acquired resistance to therapy in 6–8 months from initiation [19]. Interestingly, although the assessment of BRAF mutations nowadays is essential in clinical oncology practice, per the most recent AJCC and NCCN guidelines, only lactate dehydrogenase (LDH) is currently used for staging and prognostication, but the results can only be interpreted in advanced-stage melanoma due to LDH being a general marker of inflammation [20]. Therefore, developing a technique or biomarker to assist in the staging and early detection of recurrence is desirable to serve as a measure for treatment failure or resistance.

Differential scanning calorimetry (DSC) is a biophysical technique that directly measures the thermodynamic properties associated with temperature-induced macromolecular conformational transitions. The clinical utility of DSC has been intensely studied over the last ~15 years across many diverse diseases. The growing body of work suggests the ability to differentiate between the health statuses of patients based upon the thermodynamic properties of biofluid proteomes [21,22,23,24,25,26,27,28,29,30,31,32]. DSC provides an exquisitely sensitive way to monitor the disease state changes that affect protein thermal stability (e.g., differences in the concentration of proteins, post-translational modification of proteins, formation of biomarker–protein interactions, alterations in protein–protein networks). The composite effects of these disease-related changes on the global thermal stability profile of the biofluid proteome are captured by DSC profiles (thermograms). It is important to note that the DSC thermogram is distinct from the similarly named thermography technique. Thermography uses infrared imaging to detect differences in the surface temperature of breast tissue and is based on the principle of higher metabolic activity and vascular circulation associated with cancerous tumor development [33]. We are not aware of the use of thermography in melanoma. Conversely, a thermogram utilizes DSC to detect temperature-induced conformational changes of proteins in biofluid specimens. In the melanoma setting, three previous studies evaluated DSC as an analytical tool to delineate healthy controls and patients with active disease [22,23,24]. These studies demonstrated a proof of concept for the application of DSC for the diagnosis and monitoring of melanoma patients. However, the three studies were small pilot studies; the studies from the Lőrinczy group [23,24] analyzed five healthy controls and 15 patient samples in one study and 10 healthy controls and 35 patients in the second study, with an observation of changes in thermogram appearance according to melanoma stage (Clark’s level and Breslow’s depth) and metastasis. In our previous work [22], there was also a low number of patients (10 patients), but the study was expanded to include the longitudinal analysis of patients (63 total plasma samples from 10 patients). Although multiple thermogram parameters were evaluated, a single value calculated from the combination of three thermogram parameters, the so-called “d-value”, was used as a diagnostic parameter for the longitudinal study, with comparison to radiological and clinical assessment [22]. When analyzing longitudinal changes in d-values for the melanoma patients under surveillance, the values were generally concordant with the clinical assessment of the disease. However, the limitations of this study were the evaluation of a small number of patients and the fact that the majority of the analyzed samples were collected during treatment, which could have had an impact on thermograms.

In this manuscript, we build upon the findings of our previous work [22] by examining 23 thermogram parameters in plasma samples obtained from a larger cohort of participants who were divided into three subgroups (healthy control subjects, melanoma patients with no evidence of disease (NED), and melanoma patients with active disease). The study aimed to: (1) validate the utility of thermograms for screening and follow-up of melanoma patients, and (2) identify which of the thermogram parameters performed best and may potentially be useful for assessing prognosis. We envisioned that DSC may complement established diagnostic techniques such as radiological imaging and may improve follow-up and prognostic evaluation of patients. We hypothesize that DSC may provide earlier detection of circulating biomarkers related to melanoma metastasis/progression before changes are observed via radiological imaging. Moreover, since DSC only requires a blood draw, it would be less invasive and more cost-effective than imaging modalities. This may allow more frequent patient testing which could potentially result in earlier detection of changes in clinical status and improved patient management.

## 2. Materials and Methods

This manuscript describes a baseline study that evaluated the potential utility of thermograms in the diagnostic and prognostic assessment of melanoma among patients attending University of Louisville clinics that were not actively receiving treatment. To test the efficacy of DSC analysis, clinical reference data were compared to the thermogram parameter data. Each plasma sample was obtained at a specific clinical visit and had an accompanying clinical designation of NED or active status. Some of these designations had accompanying status determinations (e.g., radiological findings) that validated the status designations. Assumptions were made that if a patient had a recurrence, subsequent plasma samples were designated as active unless further imaging or clinical reasoning designated otherwise. 

Only samples that fulfilled the following inclusion and exclusion criteria were analyzed: (i) only one sample per subject was included (where more than one sample was available, the earliest chronological sample was selected as long as it fulfilled the other inclusion criteria), (ii) for melanoma patients in the active group, specimens were collected either before any treatment began (chemo/radio/immunotherapy) or at least 28 days after the last treatment cycle ended, (iii) for NED melanoma patients, specimens were collected at least 28 days after treatment finished and were included if no recurrences were recorded for at least 180 days after specimen collection, (iv) control subjects could not have active malignancies; also, subjects with any past history (when available) of melanoma were excluded. After applying the sample inclusion and exclusion criteria, 107 of the 108 plasma samples were from melanoma patients with White ethnicity and one sample was from a patient with non-White ethnicity. The single non-White patient sample was excluded from analysis, as ethnicity was found to affect thermograms in our previous study [26]. The ethnicity of the control group was matched to the ethnicity of melanoma patients.

### 2.1. Patient Population

The study was reviewed and approved by the Institutional Review Board at the University of Louisville (IRB# 08.0388, 10.0144, 14.0517) in compliance with the Declaration of Helsinki. De-identified plasma samples and associated patient data were obtained from the Clinical Trials Office Biorepository of the James Graham Brown Cancer Center. For each sample, information about patient status (control, active melanoma, NED melanoma), clinical data, and demographic status were provided and used for analysis. Healthy control samples were either obtained from a commercial source (8 specimens; Innovative Research, Novi, MI, USA) or were collected from patients with clinically confirmed benign lung nodules attending the surgical oncology, thoracic oncology, and pulmonology clinics at the University of Louisville who were verified as having no active cancers. 

### 2.2. Collection and Storage of Plasma Samples

Plasma specimens were obtained by collecting blood into 5 mL green-top vacutainers containing sodium heparin. Vacutainers were immediately processed as follows: (1) gently mixed via inversion 8–10 times, (2) centrifuged at 1163× *g* for 10 min, (3) the upper plasma phase was carefully aspirated to avoid hemolysis or contamination of the separated blood phases, and (4) plasma was aliquoted and immediately stored at −80 °C until analyzed via DSC. We have previously evaluated the effect of plasma sample storage conditions on DSC results and found no significant effect on thermograms for various storage times, temperatures, and freeze–thaw cycles, with the exception of storage of more than two weeks at 4 °C [34]. Patient characteristics represent those of the patient populations attending University of Louisville clinics.

### 2.3. DSC Sample Preparation and Thermogram Collection

The processing of plasma samples was performed as previously described [22,31]. Briefly, all samples were dialyzed for 25 h at 4 °C against a buffer consisting of 1.7 mM KH_2_PO_4_, 8.3 mM K_2_HPO_4_, 150 mM NaCl, 15 mM sodium citrate, pH 7.5, followed by filtration through 0.45 µm filters (Pall Corporation, New York, NY, USA). Before DSC analysis, samples were diluted 1:25 using filtered buffer (0.2 µm, Pall Corporation, New York, NY, USA) from the last step of dialysis. The same buffer was also used as a reference solution for DSC analysis. The exact protein concentration of plasma samples was determined using the bicinchoninic acid protein assay kit following the microplate protocol (Pierce, Rockford, IL, USA) and a Tecan Safire plate reader (Tecan U.S., Research Triangle Park, NC, USA).

DSC data were collected as previously described [22] using a Nano DSC Autosampler System (TA Instruments, New Castle, DE, USA). The instrument was serviced and interim performance was evaluated, following manufacturer protocols. Samples and dialysis buffer were loaded into 96-well plates and placed in the DSC instrument autosampler at 4 °C until analysis. The analysis of each sample involved a pre-scan equilibration step of 15 min at 20 °C, followed by data collection during sample heating at a scan rate of 1 °C/min over a temperature range of 20 °C to 110 °C. Duplicate DSC scans were collected for all samples.

DSC data were post-processed using Origin 7 (OriginLab Corporation, Northampton, MA, USA), as previously described [22]. The process involves subtracting a buffer reference scan from the raw sample scan, normalization for total protein concentration, and correction for non-zero sample baselines by applying a linear baseline fit. All data are presented as the average of duplicate DSC scans and plotted as excess specific heat capacity (cal/°C.g) versus temperature (°C), with final analysis performed in a temperature range of 45 to 90 °C at a step of 0.1 °C. Thermogram data for all patient samples included in this study can be found in Table S1 to encourage use by other researchers; these data are also available through GitHub via the link provided in the Section 2.4.

### 2.4. Statistical Analysis

Thermograms were evaluated in the temperature range of 45–90 °C. All statistical analyses were performed using R software [35]. R functions were developed to calculate thermogram summary metrics, including the following: thermogram peak width at half height (Width), total area under the thermogram (Area), maximum peak height (Max), median heat capacity (Median), temperature of the peak maximum (T_Max_), maximum excess specific heat capacity (C_p_^ex^) of the first peak in the region 60–66.9 °C (Peak 1), maximum C_p_^ex^ of the second peak in the region 67–72.9 °C (Peak 2), maximum C_p_^ex^ of the third peak in the region 73–78 °C (Peak 3), minimum (valley) between Peak 1 and 2 (V1.2), the position of Peak 1 (T_Peak 1_), Peak 2 (T_Peak 2_), Peak 3 (T_Peak 3_), and V1.2 (T_V1.2_), as well as the Peak 1/Peak 2 ratio (Peak 1/2), Peak 1/Peak 3 ratio (Peak 1/3), Peak 2/Peak 3 ratio (Peak 2/3), V1.2/Peak1 ratio (V1.2/Peak 1), V1.2/Peak2 ratio (V1.2/Peak 2), V1.2/Peak3 ratio (V1.2/Peak 3), and the first moment temperature (TFM), which are depicted in Figure S1. Two methods were implemented for identifying peaks. The first method evaluated peaks using the findpeaks function from the R package pracma [36], which identifies regions of monotonic behavior with a change point. Thermogram regions where excess heat capacity increased at three or more temperature steps, followed by three or more decreases, were identified as peaks. The second method for peak identification was to set a pre-defined temperature region (indicated above), of which the maximum value of the interval was identified as the peak position. Valleys were identified by finding the minimum value between any two peaks. Both peak-finding procedures resulted in identical positions and amplitudes of Peaks 1 and 2, with small differences in the choice of Peak 3. Because T_Peak 3_ was identical for the majority of samples, this parameter was not included in the statistical analyses. The agreement between these two methods, along with prior use in other publications [37,38], confirmed the proper identification of Peaks 1 and 2. Although the peak-finding functions did not identify peaks that present as shoulders, functions are continuing to be improved for the robust identification of important thermogram features. R functions for generating summary information from plasma thermograms are available at https://github.com/BuscagliaR/tlbparam (deposited on 14 December 2022). Additionally, to use the full information from the thermogram, principal components (PCs) were calculated from the primary thermogram data. PCs were calculated using the standard R function *prcomp*, using all thermogram readings from 45 to 90 °C. The first 5 PCs explained 97.7% of the variation in the thermogram measurements.

GraphPad Prism 9 software (GraphPad, La Jolla, CA, USA) was used for data visualization using boxplots with whiskers indicating 5th and 95th percentiles, and points falling outside of this range were labeled as dots. All remaining graphs were prepared using R software. Statistical modeling was performed to evaluate differences in the mean (or median) thermogram summary metric/PC values depending on patient status. Modeling was performed for each summary metric/PC using patient status and sex as covariates. Model selection procedures were implemented to reduce complexity based on partial F-tests, starting from an interaction model of sex and status and reducing until significant models were obtained. The normality of the residual distribution of each summary metric/PC and the homogeneity of variance were visually assessed using quantile–quantile plots and plots of residual versus fitted value, respectively. Any chosen models that did not pass normality assumptions were re-evaluated using quantile regression implemented via the rq function in the quantreg package [39]. Patients’ sex was included as a covariate given the known sex differences related to melanoma development, progression, and response to therapy. To determine the significance of the linear models, overall regression was evaluated using ANOVA testing. Overall regression *p*-values were adjusted for multiple comparison by applying the false discovery rate (FDR) method, using the p.adjust function in R. Final models with p.adj. < 0.05 were selected for pairwise comparisons between status groups dependent on sex (when included in the final model), using the estimated marginal means method (emmeans package [40] in R) with Tukey correction.

To differentiate between active and NED groups, logistic regression was implemented using the glm function in base R. Classification was performed using the status variable as a binary response, subdivided into NED and active groups (control patients were omitted from the logistic analysis). To improve the performance and interpretability of the models, variance inflation factors (VIFs) were calculated to reduce the multicollinearity to be no more than a VIF of 5, corresponding to no more than 80% multicollinearity between predictors. Stepwise model strategies were incorporated based on the Akaike information criterion (AIC) and Bayesian information criterion (BIC). Forward and backward stepwise models were estimated using a mixture of patient demographics and VIF reduced thermogram parameters. Selected models were validated using 25 repeats of 5-fold cross-validation. Stratified folds were used to ensure the proper representation of NED versus active populations (33 versus 74 samples, respectively). Cross-validation of the logistic regression models was based on receiver operating characteristic area under the curve (ROC-AUC) analysis [41], with the mean AUC value reported from cross-validation.

Cox proportional hazards regression analysis was used to evaluate the association of thermogram summary metrics/PCs with patient survival. Multivariable model selection was performed via backward stepwise elimination using the BIC model criterion. Kaplan–Meier survival curves were used to visualize patient survival data dichotomized based on the median value of PC3. Restricted mean survival was calculated using the survRM2 R package [42] with the Greenwood plug-in estimator to calculate the asymptotic variance.

## 3. Results

In total, this study evaluated 49 healthy control subjects, 33 NED melanoma patients, and 74 active melanoma patients. Detailed demographic and clinical factors of the three clinical groups included in the study are shown in Table 1. Stage classification used the AJCC staging system at the time of diagnosis.

**Figure 1:**
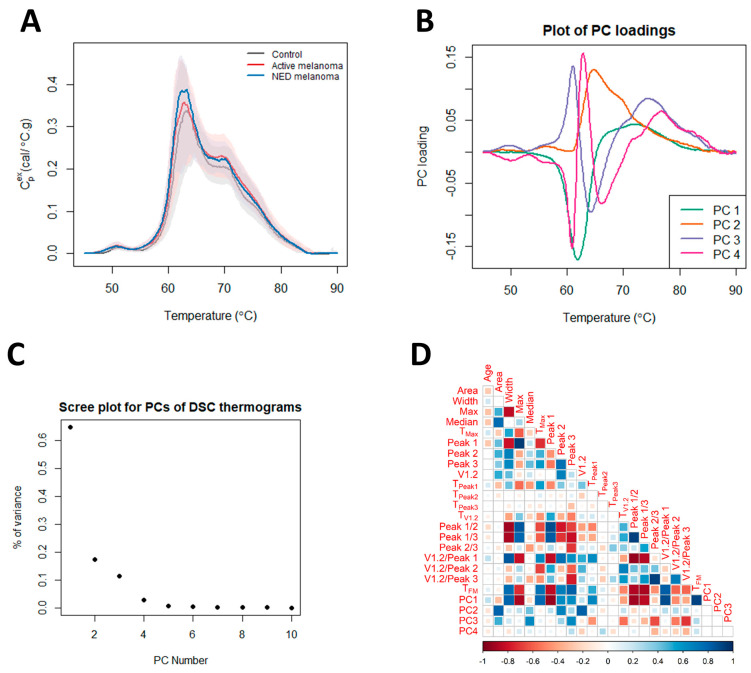
Differences in thermograms of controls, and patients with active melanoma and NED melanoma. (**A**) Plot of the median thermogram value at each temperature for active or NED melanoma and control subjects. Bands represent the 5th and 95th percentiles among subjects at each temperature. (**B**) Plot of selected PC loadings at each temperature. (**C**) Scree plot for principal components (PCs) of thermograms indicating total variance in the data, as explained by each PC. (**D**) Matrix of correlation coefficients between thermogram summary metrics and PCs.

To compare and contrast thermograms between the control, NED, and active groups, we calculated 23 parameters, 19 summary metrics, and four PCs to capture the specific features of the global protein denaturation behavior contained within each thermogram. Figure 1A shows the median thermograms and empirical 5th/95th percentiles for each of the patient status groups. The loadings for the PCs and the amount of variance explained by them are shown in Figure 1B,C. The correlation between the thermogram summary metrics and PCs is given in Figure 1D. The correlation plot (Figure 1D) provides the linear correlation estimates between each of the 23 parameters investigated. Larger filled squares indicate stronger linear relationships, with the heatmap scale given at the bottom of the image. Of note, the thermogram PCs are strongly correlated with multiple thermogram summary metrics, indicating that they represent a concise way to capture multiple thermogram features. Next, we examined multiple subgroup analyses (i.e., clinical status, cancer location, the number of affected organs, and stage) and determined the statistical performance of the 23 parameters within each subgroup. Further analysis entailed examining multiple regression models and examining various clinical variables or statistically significant parameters, as discussed below.

### 3.1. Linear Modeling of Thermogram Parameters Using Sex and Clinical Status

**Figure 2:**
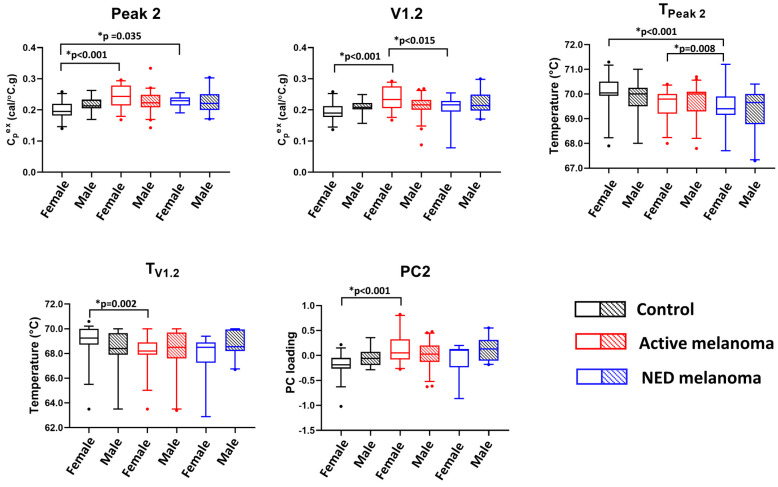
The potential utility of thermogram parameters to differentiate between controls, active, and NED melanoma patients using models with sex as a covariate and including interaction terms between clinical status and sex. Boxplots demonstrate results of a pairwise comparison for significant thermogram parameters and clinical status performed separately for each sex.

The adjusted and unadjusted *p*-values for testing the association between the thermogram parameters and clinical status while accounting for sex are given in Table S2. The boxplots in Figure 2 present significant pairwise comparisons found for various parameters and the clinical status of models, including an interaction term between clinical status and sex. Significant differences in the mean values of Peak 2, V1.2, T_Peak 2_, T_V1.2_, and PC2 were observed. Post hoc analysis indicated that for women, but not for men, the estimated measures of the center of Peak 2, V1.2, T_V1.2_, and PC2 were statistically different between the controls and patients with active disease. For T_Peak 2_, there were significant differences in parameter means when comparing the control to NED groups and NED to active groups; however, again, this was only observed for female patients.

**Figure 3:**
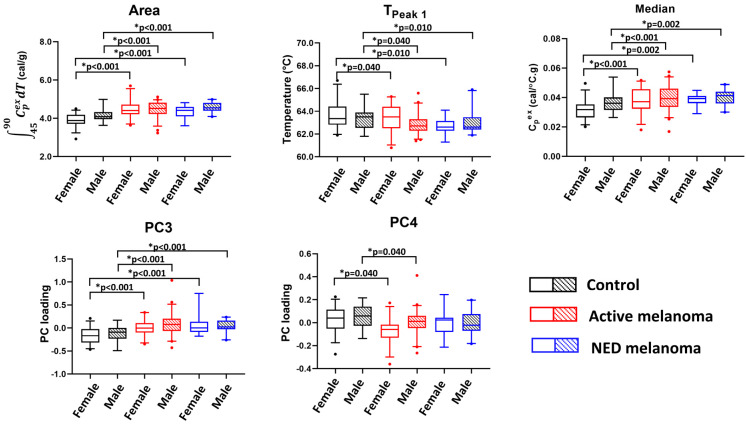
The potential utility of thermogram parameters to differentiate between controls, active, and NED melanoma patients using models with sex as a covariate and evaluating clinical status and sex as the main effects. Boxplots demonstrate results of a pairwise comparison for significant thermogram parameters and clinical status performed separately for each sex.

Five parameters were significant for models without an interaction between sex and clinical status (Figure 3). It was observed that PC4 was significantly different between the control and active patients for both females and males. Moreover, the Area, *T_Peak 1_*, PC3, and Median parameters demonstrate significant mean parameter differences between the control and melanoma patients with NED or active disease, again for both sexes. This suggests that these four parameters could be used for differentiation between control subjects and melanoma patients when used for patient classification.

### 3.2. Model Development for Classification of NED and Active Melanoma Patients

When treating melanoma patients, stringent surveillance and early detection of disease recurrence are critical in providing the most efficacious treatment plan. Therefore, we focused our attention on the development of a logistic regression model, allowing us to classify melanoma patients with active disease against NED. Logistic regression model selection returned four distinct models based on the AIC and BIC selection rules. The set of models chosen from stepwise selection are presented in Table 2. BIC selection enforces parsimonious modeling, accomplishing a desired level of prediction with as few predictor variables as possible. BIC-based selection favored highly reduced parameter selection, returning the model including only the patient age variable (model 1). Although age is an important predictor in many situations, more flexible models were sought out that focused on the use of thermogram parameters. AIC selection is less strict and forward selection resulted in the inclusion of T_Peak 2_. This parameter was selected together with age (model 2), and to evaluate the impact of using just the thermogram parameters, a secondary model using only T_Peak 2_ was investigated (model 3). Backward AIC stepwise selection produced a flexible model including age mixed with five thermogram parameters: Width, T_Peak 1_, T_Peak 2_, Peak 2/3, and PC4. This flexible model (model 4) was validated to see whether the use of this larger mixture of parameters could improve NED versus active classification.

Model validation indicates that the age variable is a key predictor of NED versus active status. The inclusion of T_Peak 2_ improved the AUC results slightly, indicating that this variable does have the ability to improve differentiation between patient status, but has a relatively low effect size based on the results presented within this work. The evaluation of the model using only T_Peak 2_ suggests that this variable does indeed differentiate between the two status types, but does so with less of an impact than when the age variable is included as a covariate. The increased flexibility model (model 4) that uses several thermogram parameters in tandem with age was comparatively worse when validated than using the covariates age and T_Peak 2_.

A more robust breakdown of the sensitivity and specificity results are presented in Figures S2 and S3. As classification metrics such as sensitivity and specificity are reliant on a predicted class probability cutoff, AUC provides an overview of classification performance by evaluating across all possible cutoffs. Figure S2 presents the mean AUC curve for all cross-validation iterations and a 95% prediction interval. Figure S3 provides the dispersion of AUC, accuracy, sensitivity, specificity, and balanced accuracy, calculated at a probability threshold of 0.5. Figure S2 demonstrates that only small differences were observed in AUC results, as presented in Table 2. Figure S3 provides the differences in sensitivity and specificity at a given threshold, with the more parameterized model 4 showing the highest balanced accuracy. All of the models favor a strong specificity for predicting active melanoma given that the patient has an active melanoma, but lack in sensitivity, providing relatively high false positive rates.

The final selected model (model 2) is given in Table 3 along with the estimated coefficients and resulting interpretations. The model with age and T_Peak 2_ shows the strongest AUC cross-validated performance and gives useful insights into how the shift in the Peak 2 temperature relates to the presence of active melanoma.

To produce a discussion related to the probability of NED versus active, predictions were made based on median age and median T_Peak 2_ values observed in the collected patient data (median age of 57 and median T_Peak 2_ value of 70 °C). Using these values within model 2 suggests a probability of 74.1% (95% confidence interval (CI): (64.1, 84.0)) for having active melanoma versus NED. To evaluate the effect of changing age or T_Peak 2_, we allow these variables to decrease by one standard deviation, as observed within the data set. Reducing age by one standard deviation to a value of 43 and keeping T_Peak 2_ constant at the median value of 70 °C suggests a probability of 61.1% (95% CI: (44.2, 76.3)) for having active melanoma versus NED. To put the impact of T_Peak 2_ into the perspective of that of age, reducing the T_Peak 2_ variable by one standard deviation to a value of 69 °C and maintaining age at the median value of 57 gives an estimated probability of 63.4% (95% CI: (51.0, 75.7)). This shows that one standard deviation changes in age and T_Peak 2_ have a similar impact for differentiating between NED and active melanoma.

The large discrepancy between the two variables’ odds-based interpretation is due to the difference in a unit change in age versus a unit change in T_Peak 2_. Within the data set, the T_Peak 2_ value only ranges from 67.3 to 71.2 °C, approximately 4 units of variability in the extreme observations, while age ranges from 23 to 93. Therefore, a unit change in age is commonly observed, while a unit change in T_Peak 2_ would be an extreme effect. However, when normalized to a standard deviation shift in age or T_Peak 2_, we see a relatively equivalent impact on the probability of active melanoma. As such, T_Peak 2_ seems to be an important predictor of active status and should be further studied for its utility in differentiating between NED and active forms of melanoma.

### 3.3. Mean Thermogram Parameters as Modeled According to Cancer Location, Number of Affected Organs/Tissues, and Clinical Stage

For patients with active disease, subgroups were made to evaluate the potential linear relationships between thermogram summary metrics/PCs and cancer location, and the number of affected organs/tissues and clinical stage, as indicators of disease advancement and prognosis. Cancer location was divided into localized (skin, lymph nodes, eye) versus distant metastasis (lungs, liver, pancreas, brain, abdomen, small intestine, head and/or neck, bones, spleen, adrenal gland, limbs). The number of affected organs/tissues was divided into groups “1”, “2”, and “greater than 2”. For the clinical stage, the samples were divided into stage 3 and stage 4, with one sample of stage 2 excluded from the analysis. Statistical analysis was performed in the same manner as presented for the thermogram parameters with clinical status (Section 3.1), and we included sex as a covariate. Unfortunately, no statistically significant differences in the mean thermogram summary metrics/PCs were found between any of these subgroups; however, for some parameters, unadjusted *p*-values below 0.1 were observed: Max, Peak 1, and Peak 1/2 presented different mean values when modeled against the number of affected organs/tissues, and there were mean differences in V1.2/Peak 2 when modeled against clinical stage and mean differences in Max when modeled against cancer location. The findings from this study do not show a linear relationship between individual thermogram parameters and clinical characteristics of melanoma, although these results will continue to be validated with increased sampling. The results of the analysis can be found in Table S3.

### 3.4. Thermogram Parameters for Modeling Overall and Progression-Free Survival of Melanoma Patients

**Figure 4:**
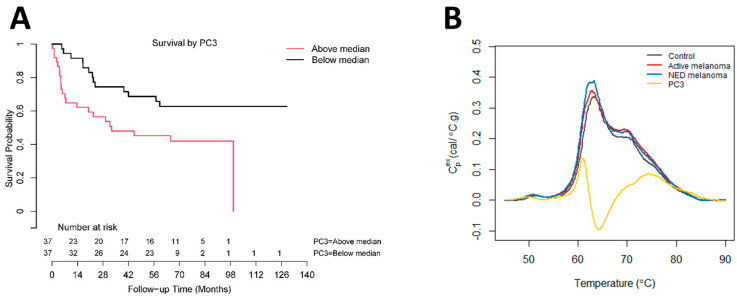
Association between overall survival of active melanoma patients and PC3. (**A**) Analysis of survival functions for overall survival of active melanoma patients dichotomized by the median value of PC3, using the Mantel–Cox test (*n* = 74). (**B**) Overlay graph of thermograms for each clinical group and PC3 loadings.

Using Cox proportional hazards regression analysis, thermogram summary metrics/PCs were evaluated for their use as predictors of progression-free survival (PFS) or OS of melanoma patients. OS analysis was performed for active melanoma patients with the time counted from the day of sample collection. The results of Cox survival analysis can be seen in Figure 4 as well as Table 4 and Table S4. Table S4 provides the *p*-values (unadjusted and adjusted) for each parameter from the univariate analyses. The most significant result was for PC3 (unadjusted *p*-value = 0.007, FDR-adjusted *p*-value = 0.13). Backward selection based on the BIC and starting with PC3, sex, and the V1.2/Peak 2 ratio resulted in only PC3 being retained in the end model. The Cox model parameter estimates for PC3 are presented in Table 4 (hazard ratio (HR) = 1.74; 95% CI: (1.17, 2.58)). The Kaplan–Meier OS curve for PC3 was constructed by splitting the data into two groups above and below the median value for PC3 (Figure 4A). The curve demonstrates lower OS for patients with PC3 values above the median. A restricted mean survival (using a truncation time of 8 years) of 4.1 years (95% CI: (3.0, 5.2)) was obtained for patients above the median PC3 value, with a restricted mean OS of 5.8 years (95% CI: (4.9, 6.8)) for patients at or below the median PC3 value (difference = 1.7 years, 95% CI: (0.245, 3.2), *p* = 0.023). Through an inspection of the PC loading plot (Figure 4B), subjects with higher PC3 values likely had wider secondary peak tails at >70 °C and a deeper valley between Peak 1 and Peak 2. This was corroborated by the high positive correlation between PC3 and the thermogram summary metrics Median and Peak 3, and the negative correlation between Peak 2/3 and V1.2/Peak 3 (Figure 1D).

PFS analysis was performed for the NED group only, as many of the active disease patients had samples collected after an initial recurrence. Of the 32 NED patients with PFS data, 7 experienced recurrences. Similar to the analysis of OS, time was counted from the day of sample collection. Table S5 includes the results of the univariate analysis. No thermogram summary metrics/PCs had adjusted *p*-values below 0.1.

## 4. Discussion

The purpose of the current study was to build upon previous work toward using DSC as a biomarker for melanoma [22] that could be especially useful for the surveillance of patients with advanced melanoma, where cancer cells had metastasized to lymph nodes or other organs. For these patients, existing diagnostic techniques like dermatoscopy or radiological imaging might not be accurate or sensitive enough to detect metastases, and the use of radiological imaging might be limited by availability, expenses, or radiation burden. Our previous work in the lung cancer setting concluded that although several thermogram parameters are correlated with clinical characteristics, more complex thermogram-based algorithms are required to adequately characterize differences in thermogram parameters between control and cancer patients [31]. Similar observations were noted by other researchers applying DSC to the classification of lung cancer and pancreatic cyst patients. These studies found that although individual thermogram parameters exhibited good performance in distinguishing between lung cancer patients and control subjects with benign lung nodules, ultimately, a combination of parameters provided superior diagnostic performance [30,32]. In the current work, linear models were constructed that modeled changes in thermogram parameters against clinical status. Several thermogram parameters (Peak 2, T_Peak2_, V1.2, T_V1.2_, and PC2) were found to have measures of center that were significantly different between at least two groups, although pairwise comparisons only revealed differences in the female subgroups. Other thermogram parameters (Area, T_Peak 1_, PC3, Median, and PC4) demonstrated significant differences between at least two clinical groups for both females and males that could be used to differentiate between the control and the melanoma (NED and active) patient populations. Yet, NED and active comparisons within the sex-included models suggested no statistical significance, which prompted us to evaluate more complex models for thermogram-based clinical differentiation.

Logistic regression analysis focused on the classification of melanoma patients with NED and active status, and found that the age and T_Peak 2_ parameters were key predictors of patient status. As seen in Table 3, odds-based analysis for T_Peak 2_ and age show only minor odds changes for age, while T_Peak 2_ has a 65% increase in the odds of having active melanoma per a 1 °C shift. Although this impact seems very large, when scaled to a one standard deviation change based on the observed ages and T_Peak 2_ within the data set, the relative change in the probability of having active cancer is nearly equivalent. This demonstrates that T_Peak 2_ seems to be an important predictor of active status and should be further studied in a larger cohort to evaluate its efficacy for the classification of NED and active forms of melanoma and to assess the utility of DSC for the surveillance of melanoma patients as a complementary technique to radiological imaging.

To further assess the performance of thermogram parameters, this study evaluated the utility of DSC to predict the OS and PFS of melanoma patients. Through OS analysis, we investigated all 23 thermogram parameters using adjusted and unadjusted *p*-values with both univariate and multivariate Cox analysis. For the univariate analysis, PC3 was the most significant parameter with an FDR-adjusted *p*-value of 0.13. The estimated restricted mean survival was 1.7 years higher for patients below the median PC3 value. PC3 also demonstrated significance in differentiating between healthy controls and both NED and active melanoma patients. This analysis is promising and proposes that PC3 could be used as a potential biomarker for patients with active melanoma to predict OS. Since the thermogram PCs are highly correlated with other thermogram summary metrics, they also provide concise measures to capture multiple features of thermograms which are related to patient outcomes, without the need to test each summary metric individually. PFS analysis demonstrated no statistical differences for any of the thermogram parameters in predicting recurrence in NED melanoma patients. It is interesting to note that prior studies have found different thermogram parameters as being significant for both OS and PFS. For patients with lung cancer, T_Peak 2_ was significant for OS, with T_Peak 1_, Peak 2/3, and PC2 having some association at lower significance levels [31], whereas in glioblastoma, the prediction of OS was associated with Area [43]. For PFS, T_Max_ was found to be a predictor of PFS in glioblastoma and lung cancer patients [31,43]. These parameters were not significant in this current study, which suggests that different thermogram parameters could be significant for different cancers.

These observations are similar to the reports that the most significant thermogram parameters for clinical classification are not necessarily retained from one study to another, and ultimately, models utilizing a combination of multiple thermogram parameters were found to have the highest diagnostic performance [30,32]. Prior studies have investigated the use of DSC for the detection of recurrence or correlating particular features of thermograms with response to a specific cancer treatment [22,44,45]. While promising, these studies were preliminary conducted with small sample sizes and the observations were never confirmed using larger, independent cohorts of patients. Unfortunately, the current study examined patients undergoing non-homogenous treatment regimens that were often changed when progression was detected. Therefore, we were unable to build a model allowing for the prediction of treatment outcome (response, stable disease, or progression) or adverse response of patients to treatment using thermogram data. Our study was also limited by the observational nature of our data and the samples available in our institution. This prevented differentiation between active and NED melanoma stratified by subgroups, including cancer stage, sex, ethnicity, and age. Within the past decade, targeted therapies (e.g., BRAF inhibitors or PD-1 immune checkpoint inhibitors) have greatly changed the landscape for the treatment of advanced melanoma. Yet, there are still therapeutic challenges [46]. Given the identification of thermogram parameters with potential utility for differentiation between clinical status and prediction of OS, we are interested in examining the use of thermogram parameters to predict treatment outcomes in a future study with controlled treatment groups, which could be helpful in guiding a clinician’s decision about initial therapy choice or change in treatment protocol if a patient is developing acquired resistance.

The continued study of thermograms for disease characterization has demonstrated ongoing potential utility in differentiating between clinical states. However, the analysis of thermograms is complex and requires further development to confirm how thermogram information can improve patient diagnostic and prognostic models. Across the multitude of studies using DSC as a clinical tool, there are notable differences in how thermograms are processed and the statistical analysis is employed [28,29,32,47]. This suggests that more work is needed to evaluate thermogram experimental and statistical methodologies and ensure the rigorous clinical use of thermograms across studies. The methodology presented in this paper is consistent with our prior studies [37,38] and uses the interpolation of raw data to ensure comparable heat capacity measurements on an equivalent temperature mesh. A consistent statistical/mathematical evaluation of thermograms is also essential in the objective quantification of the differences between clinical groups, and we provide the R functions used for the calculation of thermogram summary measures through a publicly available GitHub repository. Peak and valley identification is an area of limitation with a need for continued improvement, specifically regarding the identification of inflection points that often present as shoulders rather than distinct peaks. Estimates of thermogram peaks and valleys are reinforced through the use of two different approaches, maximum value in a temperature window and functional derivatives, with each providing consistent and reproducible estimates for Peak 1 and Peak 2. The peak-finding methods presented here are unchanged from earlier publication work to ensure consistency across studies. The ultimate goal is to develop algorithms incorporating thermogram summary measures into clinical decision tools for patient diagnoses and prognoses.

## 5. Conclusions

This study further demonstrates that DSC can be used as an investigational tool for patients with melanoma. Several thermogram parameters within ANOVA modeling were found to have significantly different measures of center between the controls and melanoma patients. Developed classification models elucidate the impact of thermogram parameters and the potential for generalized linear models to use mixtures of patient meta-information and thermogram parameters to improve the classification of patient status. Interestingly, we found that PC3 was specific enough to be used as a single biomarker for the prediction of OS in active melanoma patients and for differentiation between control individuals and melanoma patients. Additionally, we used logistic regression to classify NED and active melanoma patients, which demonstrated that thermograms could be used for the detection of recurrence during patient surveillance. Logistic regression models found T_Peak 2_ to be a significant differentiator of patient status, with increases in T_Peak 2_ corresponding to increased odds of having an active status. Further analysis could examine the predictive accuracy of the thermogram parameters and models discussed in this work. Ultimately, DSC appears to be a promising complementary technique for disease characterization, but continued rigor must be given to ensure the replication of clinical DSC results and continued validation of statistical modeling in larger studies.

## Figures and Tables

**Table 1:** Demographic and clinical characteristics of control subjects and melanoma patients included in the DSC analysis.

	Control	Active Melanoma	NED Melanoma
N	49	74	33
Age at diagnosis: median (range)	59 (25–93)	62 (26–93)	53 (23–79)
Sex:			
Female	32 (65.3%)	27 (36.5%)	13 (39.4%)
Male	17 (34.7%)	47 (63.5%)	20 (60.6%)
Ethnicity:			
White	49 (100%)	74 (100%)	33 (100%)
**Melanoma cancer classification**			
Stage:			
1		0 (0%)	0 (0%)
2		1 (1.4%)	12 (36.4%)
3		18 (24.3%)	21 (63.6%)
4		55 (74.3%)	0 (0%)
Number of affected organs/tissues:			
1		33 (44.6%)	
2		19 (25.7%)	
≥3		22 (29.7%)	
Cancer location:			
Localized		33 (44.6%)	
Distant		41 (55.4%)	

**Table 2:** Summary of model selection parameters used for classification of NED and active melanoma patients. The mean AUC is reported based on 25 repeats of fivefold stratified cross-validation.

Model	Model Variables	Selection Method	Mean AUC
1	Status ~ age	BIC	0.6563
2	Status ~ age + T_Peak 2_	Forward AIC	0.6629
3	Status ~ T_Peak 2_	Hand-selected	0.6306
4	Status ~ age + Width + T_Peak 1_ + T_Peak 2_ + Peak 2/3 + PC4	Backward AIC	0.6381

**Table 3:** Estimated model coefficients, standard errors, and percentage change in odds. The estimated coefficients are for model 2, including the two covariates age and T_Peak 2_. The table gives interpretations on the odds scale, indicating that a unit change in age will relate to 4.4% increased odds of having active melanoma. The T_Peak 2_ variable shows a large impact on the odds ratio, with a unit increase (1 °C shift in T_Peak 2_) relating to 65% increased odds of having active melanoma.

Coefficient	Estimate	Standard Error	Odds-Based Interpretation
Age	0.0427	0.0166	4.4%
T_Peak 2_	0.5010	0.3001	65.0%

**Table 4:** Summary of model parameter estimates for PC3 obtained via Cox proportional hazards regression analysis of active melanoma patient overall survival.

Model Estimates for PC3			
Parameter	Parameter Estimate	Hazard Ratio	95% Confidence Interval
PC3	0.553	1.74	(1.17–2.58)

## Data Availability

DSC data are available as Supplementary Materials.

## Supplementary Materials

The following supporting information can be downloaded at https://www.mdpi.com/article/10.3390/curroncol30070453/s1: Figure S1: Selected thermogram features evaluated in the study; Figure S2: ROC curves representing the results of 25 iterations of fivefold cross-validation for (A) model 1, (B) model 2, (C) model 3, and (D) model 4, used for classification of NED and active melanoma patients; Figure S3: Boxplots for the dispersion of key classification metrics including AUC, Accuracy (Acc), Sensitivity (Sens), Specificity (Spec), and Balanced Accuracy (Bal.Acc) for all four models used for classification of NED and active melanoma patients; Table S1: DSC data and clinical/demographic information for all patient samples included in the study; Table S2: Summary of analysis evaluating linear models estimating changes in thermogram parameters based on clinical status with sex covariates; Table S3: Summary of analysis evaluating the association between thermogram parameters and cancer location, the number of affected organs/tissues, and clinical stage, with gender as a covariate; Table S4: Summary of univariate Cox proportional hazards regression analysis of active melanoma patient overall survival. Table S5: Summary of univariate Cox proportional hazards regression analysis of NED patient progression-free survival.
