# Supplementary material for: Plasma Thermogram Parameters Differentiate Status and Overall Survival of Melanoma Patients
Source: Curr Oncol. 2023 Jun 24;30(7):6079–96. doi: 10.3390/curroncol30070453 (PMC10378067; doi:10.3390/curroncol30070453)
Supplement: Supplementary file 1 [file curroncol-30-00453-s001.zip › curroncol-2327094 - Figure S1.pdf]

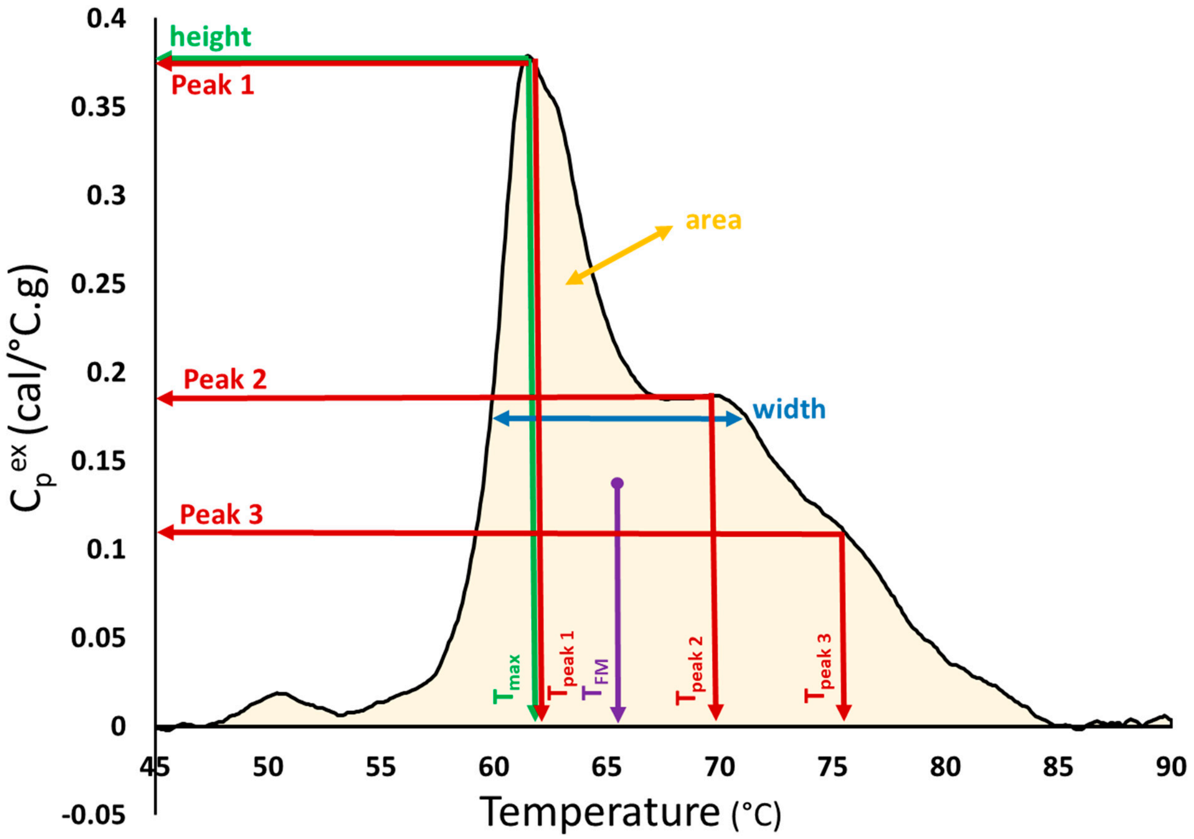

Figure S1. Selected thermogram features evaluated in the study: thermogram peak width at half height (Width); total area under the thermogram (Area), maximum peak height (Max), temperature of the peak maximum (T_{max}), first moment temperature (T_{FM}), maximum excess specific heat capacity (C_p^{ex}) of Peaks 1, 2, and 3 (Peak 1, Peak 2, and Peak 3, respectively) and the position of Peaks 1, 2, and 3 ($T_{peak\ 1}$, $T_{peak\ 2}$, and $T_{peak\ 3}$, respectively).
