# Supplementary material for: Plasma Thermogram Parameters Differentiate Status and Overall Survival of Melanoma Patients
Source: Curr Oncol. 2023 Jun 24;30(7):6079–96. doi: 10.3390/curroncol30070453 (PMC10378067; doi:10.3390/curroncol30070453)
Supplement: Supplementary file 1 [file curroncol-30-00453-s001.zip › curroncol-2327094 - Figure S2.pdf]

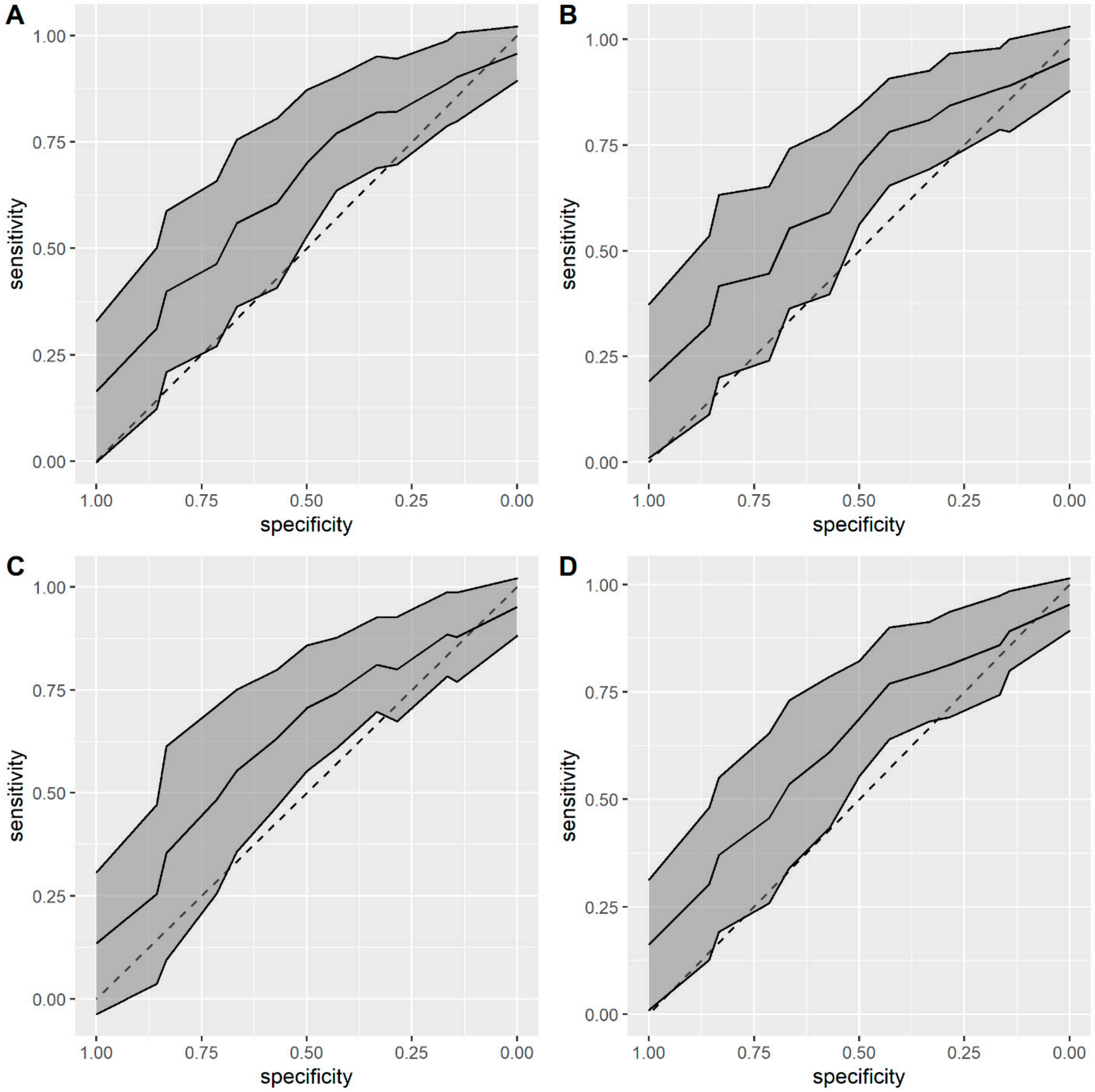

Figure S2. ROC curves representing the results of 25 iterations of 5-fold cross validation for (A) model 1, (B) model 2, (C) model 3, and (D) model 4 used for classification of NED and active melanoma patients. Each panel breaks down the sensitivity and specificity profiles across all possible predicted class probability thresholds. The plot displays the mean ROC curve and corresponding 95% confidence interval based on point-wise estimates. Minor differences are observed in the behaviors based on the covariates involved in the model.
