# Supplementary material for: Plasma Thermogram Parameters Differentiate Status and Overall Survival of Melanoma Patients
Source: Curr Oncol. 2023 Jun 24;30(7):6079–96. doi: 10.3390/curroncol30070453 (PMC10378067; doi:10.3390/curroncol30070453)
Supplement: Supplementary file 1 [file curroncol-30-00453-s001.zip › curroncol-2327094 - Figure S3.pdf]

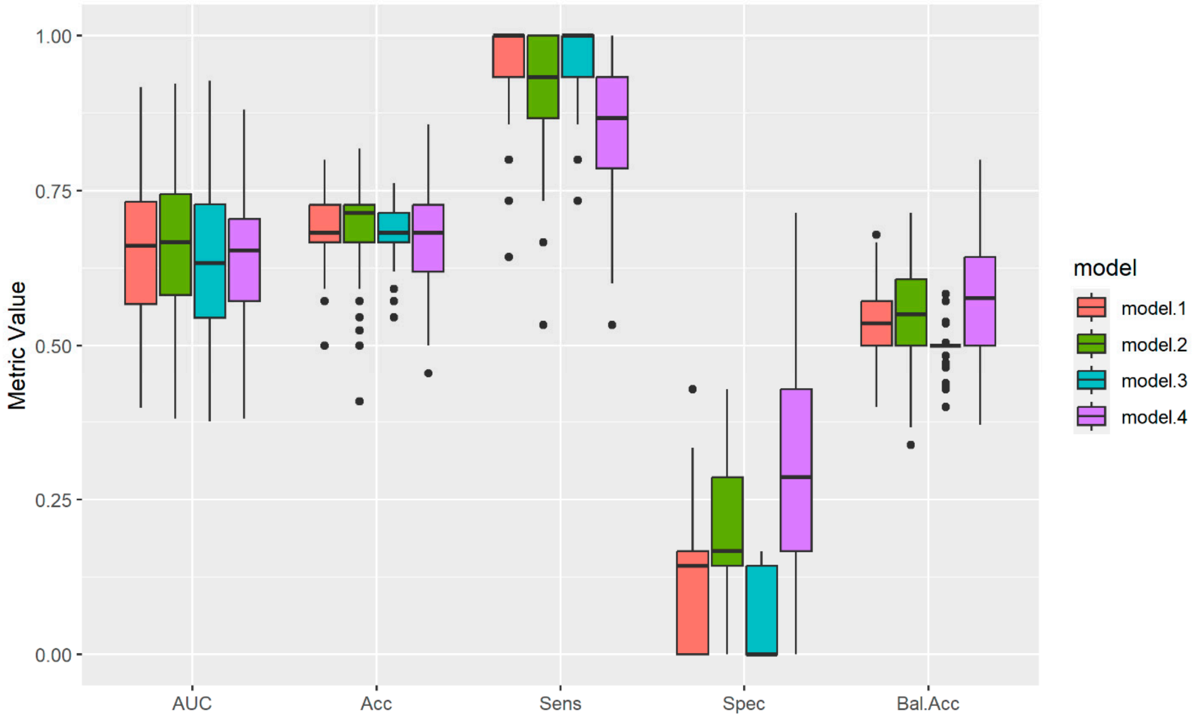

Figure S3. Boxplots for the dispersion of key classification metrics including AUC, Accuracy (Acc), Sensitivity (Sens), Specificity (Spec), and Balanced Accuracy (Bal.Acc) for all four models used for classification of NED and active melanoma patients. All models display significant dispersion in the AUC values, illustrating no single model is strongly preferred. Models 1, 2, and 3 all favor high sensitivity predictions, demonstrating the models rarely miss classifying active melanoma, but are offset by low specificity, and a tendency for false-positives. The more parameterized model 4 shows some capability to balance the predictions, providing the highest mean balanced accuracy of all models investigated, but lowering the sensitivity for accurate prediction of active melanoma.
