# Supplementary material for: Plasma Thermogram Parameters Differentiate Status and Overall Survival of Melanoma Patients
Source: Curr Oncol. 2023 Jun 24;30(7):6079–96. doi: 10.3390/curroncol30070453 (PMC10378067; doi:10.3390/curroncol30070453)
Supplement: Supplementary file 1 [file curroncol-30-00453-s001.zip › curroncol-2327094 - Table S2.pdf]

Table S2. Summary of analysis evaluating linear models estimating changes in thermogram parameters based on clinical status with sex covariates.

Parameter	Unadjusted p-value	FDR adjusted p-value
**Models with a significant sex-status interaction**		
Width	0.187	0.239
Peak 2	<0.001	<0.001
$T_{Peak\ 1}$	0.008	0.022
$T_{Peak\ 2}$	<0.001	<0.001
V1.2	<0.001	<0.001
$T_{V1.2}$	0.020	0.047
Peak 1/2	0.085	0.150
Peak 1/3	0.209	0.240
V1.2/Peak 1	0.062	0.119
PC2	<0.001	<0.001
**Models with no significant sex-status interaction**		
Area	<0.001	<0.001
Max	0.155	0.222
Peak 1	0.172	0.233
Peak 3	0.050	0.103
T_{Max}	0.124	0.204
T_{FM}	0.350	0.383
Peak 2/3	0.889	0.928
V1.2/Peak 2	0.205	0.241
V1.2/Peak 3	0.973	0.973
Median	<0.001	<0.001
PC1	0.152	0.222
PC3	<0.001	<0.001
PC4	0.006	0.016
