# Supplementary material for: Plasma Thermogram Parameters Differentiate Status and Overall Survival of Melanoma Patients
Source: Curr Oncol. 2023 Jun 24;30(7):6079–96. doi: 10.3390/curroncol30070453 (PMC10378067; doi:10.3390/curroncol30070453)
Supplement: Supplementary file 1 [file curroncol-30-00453-s001.zip › curroncol-2327094 - Table S3.pdf]

Table S3. Summary of analysis evaluating the association between thermogram parameters and cancer location, the number of affected organs/tissues, and clinical stage with gender as a covariate.

	**Cancer location**		**Number of affected organs/tissues**		**Clinical stage**	
**Parameter**	**Unadjusted p-value**	**FDR adjusted p-value**	**Unadjusted p-value**	**FDR adjusted p-value**	**Unadjusted p-value**	**FDR adjusted p-value**
**Models with a significant sex-status interaction**						
$T_{Peak\ 2}$	0.936	0.971				
Peak 2/3			0.207	0.436		
V1.2/Peak 2			0.299	0.484		
V1.2/Peak 3			0.316	0.484		
PC1	0.166	0.686				
PC2			0.195	0.436		
**Models with no significant sex-status interactions**						
Area	0.173	0.686	0.482	0.693	0.413	0.980
Width	0.812	0.929	0.866	0.906	0.737	0.980
Max	0.083	0.686	0.084	0.436	0.316	0.980
Peak 1	0.179	0.686	0.097	0.436	0.686	0.980
Peak 2	0.848	0.929	0.308	0.484	0.937	0.980
Peak 3	0.676	0.913	0.559	0.727	0.515	0.980
$T_{Peak\ 1}$	0.646	0.913	0.601	0.727	0.544	0.980
$T_{Peak\ 2}$			1.000	1.000	1.000	1.000
V1.2	0.672	0.913	0.608	0.727	0.672	0.980
$T_{V1.2}$	0.136	0.686	0.140	0.436	0.252	0.980
T_{Max}	0.971	0.971	0.288	0.484	0.656	0.980
T_{FM}	0.491	0.913	0.102	0.436	0.831	0.980
Peak 1/2	0.150	0.686	0.061	0.436	0.578	0.980
Peak 1/3	0.224	0.736	0.161	0.436	0.804	0.980

Peak 2/3	0.576	0.913			0.385	0.980
V1.2/Peak 1	0.342	0.686	0.179	0.436	0.834	0.980
V1.2/Peak 2	0.605	0.913			0.065	0.980
V1.2/Peak 3	0.742	0.913			0.432	0.980
Median	0.487	0.913	0.691	0.757	0.806	0.980
PC1			0.119	0.436	0.674	0.980
PC2	0.719	0.913			0.883	0.980
PC3	0.162	0.683	0.632	0.727	0.237	0.980
PC4	0.754	0.913	0.208	0.436	0.906	0.980
