# Supplementary material for: Plasma Thermogram Parameters Differentiate Status and Overall Survival of Melanoma Patients
Source: Curr Oncol. 2023 Jun 24;30(7):6079–96. doi: 10.3390/curroncol30070453 (PMC10378067; doi:10.3390/curroncol30070453)
Supplement: Supplementary file 1 [file curroncol-30-00453-s001.zip › curroncol-2327094 - Table S4.pdf]

Table S4. Summary of univariate Cox proportional hazards regression analysis of active melanoma patient overall survival.

Parameter	Unadjusted p-value	FDR adjusted p-value
Age	0.393	0.702
Sex	0.056	0.273
Area	0.793	0.881
Width	0.799	0.881
Max	0.830	0.881
Peak 1	0.339	0.653
Peak 2	0.789	0.881
Peak 3	0.066	0.273
TPeak 1	0.135	0.374
TPeak 2	0.579	0.852
V1.2	0.112	0.351
TV1.2	0.272	0.619
TMax	0.782	0.881
TFM	0.191	0.477
Peak 1/2	0.843	0.881
Peak 1/3	0.431	0.719
Peak 2/3	0.028	0.178
V1.2/Peak 1	0.846	0.881
V1.2/Peak 2	0.016	0.130
V1.2/Peak 3	0.013	0.130
Median	0.077	0.274
PC1	0.522	0.815
PC2	0.297	0.619
PC3	0.007	0.130
PC4	0.881	0.881
