# Supplementary material for: Plasma Thermogram Parameters Differentiate Status and Overall Survival of Melanoma Patients
Source: Curr Oncol. 2023 Jun 24;30(7):6079–96. doi: 10.3390/curroncol30070453 (PMC10378067; doi:10.3390/curroncol30070453)
Supplement: Supplementary file 1 [file curroncol-30-00453-s001.zip › curroncol-2327094 - Table S5.pdf]

Table S5. Summary of univariate Cox proportional hazards regression analysis of NED patient progression-free survival.

Parameter	Unadjusted p-value	FDR adjusted p-value
Age	0.714	0.796
Gender	0.716	0.796
Area	0.519	0.796
Width	0.652	0.796
Max	0.570	0.796
Peak 1	0.473	0.796
Peak 2	0.268	0.796
Peak 3	0.567	0.796
$T_{Peak\ 1}$	0.983	0.983
$T_{Peak\ 2}$	0.732	0.796
V1.2	0.672	0.796
$T_{V1.2}$	0.099	0.796
T_{Max}	0.106	0.796
T_{FM}	0.422	0.796
Peak 1/2	0.287	0.796
Peak 1/3	0.397	0.796
Peak 2/3	0.948	0.983
V1.2/Peak 1	0.267	0.796
V1.2/Peak 2	0.527	0.796
V1.2/Peak 3	0.653	0.796
Median	0.182	0.796
PC1	0.220	0.796
PC2	0.344	0.796
PC3	0.516	0.796
PC4	0.492	0.796
